# EPCNet: Implementing an ‘Artificial Fovea’ for More Efficient Monitoring Using the Sensor Fusion of an Event-Based and a Frame-Based Camera

**DOI:** 10.3390/s25154540

**Published:** 2025-07-22

**Authors:** Orla Sealy Phelan, Dara Molloy, Roshan George, Edward Jones, Martin Glavin, Brian Deegan

**Affiliations:** 1 Department of Electrical and Electronic Engineering, University of Galway, University Road, H91 TK33 Galway, Irelandr.george5@universityofgalway.ie (R.G.); edward.jones@universityofgalway.ie (E.J.); martin.glavin@universityofgalway.ie (M.G.); 2 Ryan Institute, University of Galway, University Road, H91 TK33 Galway, Ireland; 3 Valeo Vision Systems, Tuam, H54 Y276 Galway, Ireland; dara.molloy@valeo.com

**Keywords:** neuromorphic camera, object detection, multi-modal fusion, stereo camera

## Abstract

Efficient object detection is crucial to real-time monitoring applications such as autonomous driving or security systems. Modern RGB cameras can produce high-resolution images for accurate object detection. However, increased resolution results in increased network latency and power consumption. To minimise this latency, Convolutional Neural Networks (CNNs) often have a resolution limitation, requiring images to be down-sampled before inference, causing significant information loss. Event-based cameras are neuromorphic vision sensors with high temporal resolution, low power consumption, and high dynamic range, making them preferable to regular RGB cameras in many situations. This project proposes the fusion of an event-based camera with an RGB camera to mitigate the trade-off between temporal resolution and accuracy, while minimising power consumption. The cameras are calibrated to create a multi-modal stereo vision system where pixel coordinates can be projected between the event and RGB camera image planes. This calibration is used to project bounding boxes detected by clustering of events into the RGB image plane, thereby cropping each RGB frame instead of down-sampling to meet the requirements of the CNN. Using the Common Objects in Context (COCO) dataset evaluator, the average precision (AP) for the bicycle class in RGB scenes improved from 21.08 to 57.38. Additionally, AP increased across all classes from 37.93 to 46.89. To reduce system latency, a novel object detection approach is proposed where the event camera acts as a region proposal network, and a classification algorithm is run on the proposed regions. This achieved a 78% improvement over baseline.

## 1. Introduction

Efficient monitoring systems are essential for a wide range of applications, including autonomous vehicles and industrial surveillance. Many of these applications are safety-critical systems that require high accuracy and temporal resolution to ensure accurate real-time responses. Advancements in camera technologies enable higher-quality images, with improved resolution, color fidelity, and dynamic range.

This directly impacts improving the accuracy of object detection; however, this is at a cost to power consumption and bandwidth. Due to this, there is often a trade-off between the temporal resolution and accuracy of object detection, posing a challenge for real-time safety-critical systems. With the rise of embedded applications, the power consumption and computational complexity of these detection systems can also be restricted, and any reduction in required resources is valuable.

Many modern imaging systems take inspiration from human biology, often referred to as neuromorphic vision. In biological vision systems, the visual field of the human eye is split into two sections. The center of the field is called the fovea, and the surrounding area is called the periphery. The fovea is an area of very high visual acuity, used for recognition of highly detailed objects via cone photoreceptors. The periphery has lower resolution and uses rod photoreceptors to collect information on the overall scene [1]. Rods are more sensitive to changes in light intensity and are responsible for vision under low-light conditions. However, they have lower spatial acuity compared to cones. Conversely, cones are active at higher light levels and provide better spatial resolution.

This concept is being imitated in modern image processing systems, by implementing an ‘Artificial Fovea’ to combat the trade-off between the accuracy and latency of object detection systems. A high resolution is used to capture detailed areas of interest within an image. Conversely, the surrounding areas are captured at a lower resolution, which reduces the overall spatial detail. However, these lower-resolution areas are more sensitive to movement, enabling the system to detect changes or motion more effectively. By employing this technique, the imaging system can improve its overall temporal resolution without compromising the accuracy of the high-resolution areas.

### 1.1. Event Camera

An event camera is a neuromorphic vision sensor, which is inspired by the human vision system. These sensors are designed to process only visible changes in a scene rather than capturing the entire scene continuously. In these sensors, each pixel operates asynchronously, measuring logarithmic changes in light intensity. When a change in light intensity exceeds a predefined threshold, either above or below the previous value, the pixel records the change. This approach eliminates the need to capture redundant data from static or unchanging parts of the scene, which is a common drawback of standard RGB cameras [2]. The resulting data that are recorded by these cameras constitute a sequence of events corresponding to areas of movement in the image in the form of the *x*, *y* coordinates of the pixel, the time, and the polarity of the light intensity change (increasing or decreasing brightness).

Event cameras provide many advantages over state-of-the-art frame-based cameras. Sensors record brightness changes with microsecond resolution, achieving temporal resolutions > 10,000 fps (frames per second). The asynchronous nature of each pixel eliminates the latency of waiting for the exposure time of each frame, allowing events to be transmitted as soon as they are detected, with sub-millisecond latency. In frame-based cameras, power is wasted by recording static data in the frame. This is eliminated in event cameras, allowing them to achieve power efficiencies in the range of 10 mW. Finally, the achievable dynamic range is >120 dB compared to frame-based cameras with standard values in the range of 60 dB. This can be improved by capturing multiple images at varying levels of exposure and merging the results; however, this additional processing adds undesirable complexity to the system. It can also result in other image quality issues, such as motion blur artifacts and low signal-to-noise ratio in midtones.

**Figure 1:**
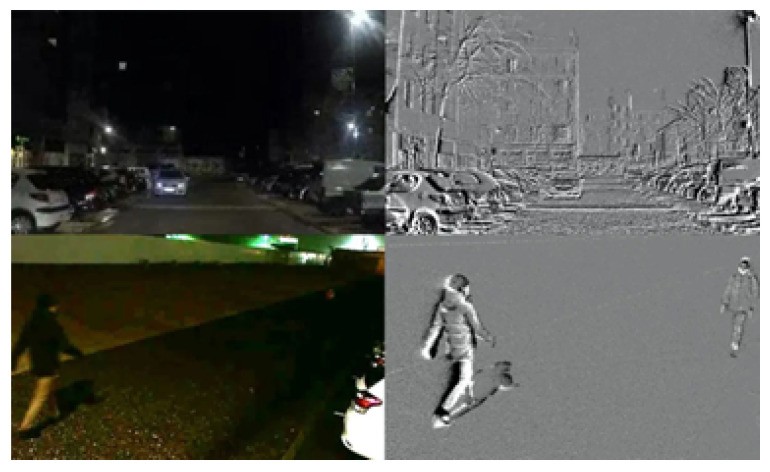
Comparison of RGB frame image versus an event camera image, taken from [2].

The high dynamic range of the event camera is achievable due to the logarithmic scale used by the pixels, and the independent nature of these pixels means that they are not constrained by the fixed exposure interval used in frame-based cameras [3]. This improved dynamic range makes event-based sensors more robust to adverse conditions such as low light or glare from the sun. Figure 1, an image taken from [2] highlights this, showing a low-light scene on a frame-based camera and an event camera. The pedestrians in the bottom image are almost undetectable in the RGB camera, while they are much clearer in the event camera due to its improved dynamic range.

At present, the resolution of state-of-the-art event cameras is lower than the resolution of standard frame-based cameras, but this high resolution may not always be a benefit. Gehrig and Scaramuzza [4] suggest that low-resolution event cameras outperform those with higher resolutions in low-illumination conditions and at high speeds. High-resolution event cameras use a higher per-pixel event rate, which causes increased temporal noise under these conditions, reducing the image accuracy. Furthermore, the Convolutional Neural Networks (CNNs) used in many object detection algorithms have a resolution limitation to meet latency constraints. The resolution of images processed by these networks is typically limited to 600 × 600 pixels or less to minimize network latency. This means that high-resolution images must be downscaled, causing significant information loss, which reduces the accuracy of the detection, especially for smaller details in the image.

### 1.2. Design Overview

The proposed solution fuses images from RGB and event-based cameras to leverage the benefits of both sensors. The RGB camera will be initalized and not capturing when there is no motion in the frame. The motion will be detected by the event camera, taking advantage of its high temporal resolution, robustness to adverse conditions, and low power consumption. This detection will trigger a signal to be sent to the RGB camera where the capture will start; therefore, no idle power will be wasted by the camera.

The event camera acts as the low-resolution peripheral vision to detect movement in the scene, while the RGB camera acts as the fovea where high-resolution analysis of the image is completed. This design will take advantage of the improved performance of the event camera at lower resolutions, leaving the high-resolution analysis to be performed on the frame-based image.

**Figure 2:**
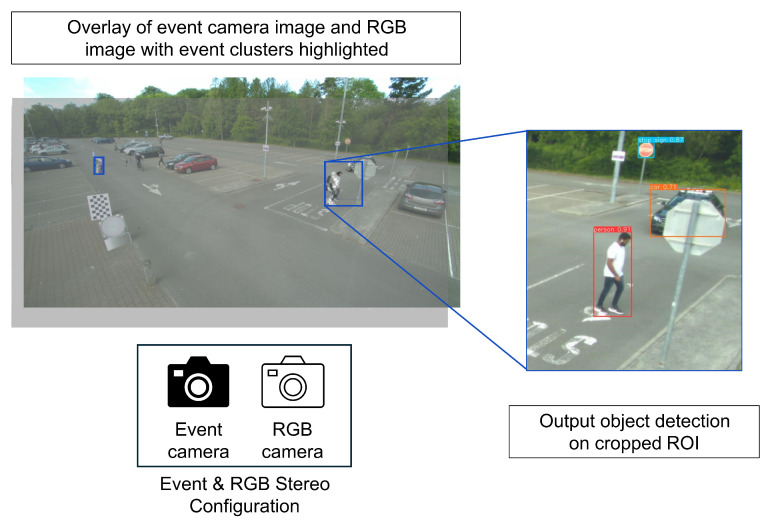
Proof-of-concept system design. The camera rig is shown with the event camera and the RGB camera side by side. This records the data of the scene in the foreground. The images from each camera are overlayed to highlight the different image planes of the cameras. The bounding boxes of the detected events, and the output object detection results that are run on the cropped ROIs of the RGB image are shown to demonstrate the steps used in the design.

The focus of this research is on the data analysis that is triggered. The proof-of-concept (POC) design is outlined in Figure 2. To implement this, the following steps are completed:

1. Perform stereo calibration between the event camera and the RGB camera to determine the relative extrinsic transformation between the two cameras.
2. Detect motion in the event camera by clustering the events.
3. Project detected clusters onto RGB image plane using the extrinsic transformation, thus determining the region of interest (ROI) in the image.
4. Crop the RGB image based on ROIs.
5. Run object detection on cropped images.

When motion is detected by the event camera, a clustering algorithm is run on the resulting data to determine the bounding boxes of the event in the event camera image plane. These bounding box coordinates are then projected to their corresponding pixel coordinates in the RGB camera image plane, determining the high-accuracy foveal areas of the image. The RGB frame is then cropped to the area of the image determined by these bounding boxes, and object detection is run on these cropped images.

By cropping the frame to these regions, the resulting images do not have to be down-sampled to meet the resolution requirements of the object detection algorithm, therefore reducing information loss. Accuracy and latency results achieved using this method are compared with the ‘out-of-the-box’ object detection method of running the same algorithm on each full frame of the RGB camera data.

The further enhancement of the system investigates the implementation of an Event-Region Proposal and Classification Network (EPCNet). The event camera clusters are used for object region proposal, and a classification algorithm is used for object classification. This EPCNet design reduces the complexity of the network by using a classification algorithm instead of an object detection algorithm, thereby reducing latency.

Analysis and testing are conducted on a computer equipped with an Intel (Santa Clara, CA, USA) Core i7-10750 CPU operating at 2.60 GHz and a NVIDIA (Santa Clara, CA, USA) Quadro T1000 with Max-Q Design graphics processing unit. Design validation was performed using the Galway Multi-Modal Infrastructure Node Dataset (G-MIND) [5,6]. The data recorded on both cameras consisted of pedestrians, cars, and cyclists moving around a car park. Individual camera specifications are highlighted in Table 1.

### 1.3. Contributions of This Paper

The main contributions of this research include the following:

- A comprehensive review of RGB and event camera sensor fusion in the literature.
- An adaptation to common camera calibration techniques that facilitates the calibration of an event-based camera with a frame-based camera using common automated software.
- A novel foveal vision framework that combines event-based and frame-based cameras to achieve the following:
  − Enhanced small object detection through selective high-resolution processing (78% accuracy improvement over baseline).
  − Reduced computational complexity via EPCNet architecture, using the event camera as a region proposer with a lightweight classifier (40% lower latency than YOLO while maintaining comparable mAP).
- A proposed system design that leverages the power efficiency of an event camera in a real-time monitoring system suitable for embedded applications.

The remaining sections of this paper are as follows. In Section 2, we discuss the recent literature in the areas of event cameras, event–RGB sensor fusion, and foveal vision to highlight the relevance of this research. Section 3 outlines the methods used in the study. It describes the technique used to stereo calibrate the two cameras and project image coordinates from one image plane to the other; the clustering and object detection algorithms used for the proof-of-concept implementation; and the final EPCNet design where the event camera acts as a region proposer for the detection of objects in the RGB image. In Section 4, the results are detailed and compared for the POC design, the EPCNet, and the baseline. The analysis of the results is focused on model accuracy and network latency. In Section 5, we discuss the results, and outline the proposed design and its advantages over the state-of-the-art. Section 6 concludes the research, highlighting some limitations of the study and introducing some future work that will be carried out to further enhance this research.

## 2. Related Works

Previous research has analyzed the accuracy of neuromorphic sensors and discussed various techniques to improve the performance of these real-time imaging systems. From this investigation of research, there is limited literature available regarding the concept of sensor fusion between an event-based sensor and an RGB frame-based camera without increasing the complexity and power requirements of the resulting system. Therefore, this research aims to combat the issues detailed by taking advantage of the improved efficiencies of these event-based sensors, while also making use of the high-accuracy object detection possible with RGB cameras.

### 2.1. Event Camera

Event cameras provide significant efficiency improvements over regular frame-based cameras, making them suitable for applications in the autonomous vehicle industry, and the accuracy of object detection that they can achieve is a topic of much recent research.

The nature of the data recorded by these sensors presents challenges for object detection and classification. The event streams are often noisy, and the spatial resolution of the sensors is limited. Rebecq et al. propose a recurrent neural network in [7] which can be used to reconstruct high-resolution grayscale images from event streams. The resulting images show impressive quality and give good classification accuracy on off-the-shelf CNNs. The image reconstruction requires the use of a fully convolutional neural network, which adds significant computational cost to the system.

Perot et al. in [8] show that this expensive image reconstruction is not necessary, as they present a neural network that can detect objects in raw event data. Their model is assessed on both raw event data and the reconstructed grayscale images employing the network introduced in [7], demonstrating enhanced accuracy on the raw event data along with significant run-time efficiency. The implementation of an object detection model using raw event camera data is further explored in [9], where a YOLOv3-based neural network is trained on a simulated event-based dataset to detect and track faces for use in driver monitoring systems. The performance of the model in detecting and tracking faces and eyes is assessed using data collected from a PROPHESEE event camera, showed promising results. The detection results show precision and recall values in the range of 80–98%, which supports the applicability of event cameras for object detection systems.

According to Perot et al. in [8], one of the main challenges when it comes to object detection on event-based image data is the lack of available event camera datasets for deep learning model training. Clustering algorithms are unsupervised machine learning algorithms that allow networks to make predictions on unlabeled data. The use of clustering algorithms on event camera data has been investigated in many works, showing high accuracies for tracking moving objects.

An early investigation on this was carried out by Hinz et al. in [10], where classical clustering techniques were investigated, showing the efficacy of clustering algorithms for multi-object tracking on event data. Since then, the idea has been further investigated with new algorithms being developed. Mondal shows the improvements that can be made using graph spectral clustering and K-means clustering in [11,12], reducing the number of model hyperparameters, and improving the robustness to noisy event data. Although these algorithms provide good results in object tracking applications, the classification of detected objects cannot be accurately implemented using clustering algorithms.

Perot et al. and Ryan et al. in [8,9] show the viability of raw event data in object detection models; however, the availability of training data to train such models is limited, with [9] using a simulated dataset for training. The use of unsupervised clustering algorithms combats this issue in [10,11,12]. However, these algorithms do not provide sufficient information for object classification. To combat these issues, the combination of data from an event-based camera and a frame-based camera would take advantage of the benefits of each and allow common frame-based object detection techniques to be implemented and advanced using the characteristics of the event camera.

### 2.2. Event–RGB Sensor Fusion

Sensor fusion is the process of combining sensor data from multiple sources with the aim of removing any gaps in the collected data. There is a significant amount of research exploring the multi-modal fusion of RADAR or LIDAR with regular cameras to gather depth information in a scene [13,14,15,16,17]. Stereo vision is also a common feature of many imaging systems, which is the fusion of images from multiple cameras into the same coordinate system. This can be used to calculate the disparity between the two cameras, which in turn can be used to determine depth in the resulting images. Event-based cameras are a relatively new technology, and there is limited research on the multi-modal fusion of these sensors.

Some recent research explores the idea of combining frame-based cameras with event-based cameras in SLAM (Simultaneous Localization and Mapping) to take advantage of the robustness of event cameras to motion blur and low-lighting conditions. Vidal et al. implement this in [18] by integrating an event-based camera, a frame-based camera, and an IMU (Inertial Measurement Unit). Feature detection and tracking are used to calculate feature tracks for both event and regular frames, and triangulation is used to map the resulting features to their 3D points while minimizing the resulting loss function. The projection of points from one camera frame to the other using the extrinsic calibration of the cameras is mentioned, but the calibration process is not described.

A similar feature matching technique is used by Perot et al. in [8] to implement this point projection between an event-based camera and an RGB camera positioned side by side. This method uses feature mapping between the raw event data stream and the corresponding RGB frames to calculate the homography between the two cameras using the RANSAC (Random Sample Consensus) algorithm, which is then used to project pixel coordinates from one camera frame to the other. This method shows good results, but ignores the intrinsic and extrinsic camera parameters, including the distortion coefficients of the lenses, which could affect its performance in unseen situations.

The advantages of event cameras for use in SLAM are also recognized in [19,20]. Both provide benchmark datasets captured on a multi-modal camera setup. The MVSEC dataset [19] combines a stereo frame-based camera, a LIDAR sensor, and IMU, and two stereo DAVIS (Dynamic and Active Pixel Vision Sensor) cameras. The DAVIS combines an asynchronous DVS (Dynamic Vision Sensor) with a synchronous frame-based sensor on a single photodiode. This sensor provides impressive results, allowing the advantages of event- and frame-based cameras to be exploited on a single chip. Due to the combination of both sensors on a single photodiode, the resolution of the sensor is only 240 × 180 pixels, resulting in low-quality frame-based images compared to state-of-the-art cameras.

The use of the DAVIS sensor in the MVSEC dataset means that the event cameras share a coordinate plane with their corresponding frame-based image plane, which makes the calibration process easier as standard calibration can be run on the frame-based image. However, the disadvantage is the low resolution of the sensor, with both frame-based and event-based cameras achieving much higher resolutions in modern implementations.

The VECTOR dataset [20] improves this by integrating an event-based stereo camera system with a resolution of 640 × 480 with a 1.2MP frame-based stereo camera, and multiple depth and inertia sensors. The calibration and synchronization between cameras are discussed in more depth here. The cameras are synchronized using a microcontroller to send pulses to each sensor, which are used to synchronize and calibrate each clock. The intrinsic and extrinsic calibration is performed using a blinking checkerboard pattern, similarly to the method proposed by Zhang in [21]. The extrinsic calibration is manually completed by detecting checkerboard corners in the 2D image and using PnP (perspective-n-point) to map between 2D and 3D point correspondences, adding time and complexity to the calculations.

An improvement on this manual calibration is implemented in the DSEC dataset [22], which combines two 640 × 480 resolution event cameras and two 1440 × 1080 resolution RGB cameras with LIDAR and GPS sensors. Event camera calibration is completed using the E2VID algorithm by Rebecq et al. [7], available at https://github.com/uzh-rpg/rpg_e2vid (accessed on 9 July 2025) to reconstruct grayscale images of a checkerboard pattern being moved in front of the camera, which can be used in common calibration frameworks. GPS is used for time synchronization between images, which are then used to calibrate the cameras with the Kalibr toolbox [23] to estimate extrinsic parameters.

In both VECTOR and DSEC dataset validations, the extrinsic calibration is used to map points between single-mode stereo pairs or from the 2D image to the 3D world to calculate depth in images, but the projection of points between event cameras and frame-based cameras is not discussed.

This projection is implemented by Tomy et al. in [24], where an object detection model is trained to take input data from both event and RGB cameras to improve the model’s robustness to adverse conditions. The proposed method uses the intrinsic parameters of each camera, combined with the rotation matrix between camera systems, to project each RGB camera pixel onto the event camera image plane.

The resulting projection shows good improvements when training the model; however, the method to calculate this rotation matrix is not documented. The model is trained on the DSEC dataset [22] and shows good accuracy improvements for different types of data corruption, highlighting its robustness to adverse weather conditions. The multi-input architecture of the model, where it takes in frames from both camera sources, suggests an increased complexity of the network, which is not evaluated or discussed in the research. The object detection algorithm trained in [8] shows similar accuracy results using only event camera frames, therefore eliminating the need for this complex, multi-input network.

In addition to SLAM and object detection systems, the sensor fusion of event-based cameras with frame-based cameras has shown improvements in visual odometry and optical flow prediction. Chen et al. implement “the first event-based stereo visual-inertial odometry” in [25] to take advantage of the high performance of event cameras in high-motion situations. Feature tracking and matching are implemented on an event-based stereo system and a frame-based stereo system, which are used to calculate depth in the scene using point triangulation. The system is evaluated using two DAVIS sensors; therefore, event camera calibration is not necessary and can be performed using standard methods on the frame-based image, similar to [19].

The same camera system is used in [26], which combines the event frames and the regular frames from the DAVIS sensor to train an optical flow estimation model. In this work, Lee et al. propose an energy-efficient ‘Fusion-FlowNet’, which combines Spiking Neural Networks (SNNs) to process asynchronous event data, and Analog Neural Networks (ANN) for frame-based images to improve the prediction of movements between frames. The computational cost of the model is kept to a minimum due to the low resolution of the DAVIS sensor, and the use of SNNs, which have few network parameters. The model performance shows high accuracy for optical flow estimation; however, for object detection systems, the low-resolution sensors would limit the detection accuracy.

The DAVIS sensor [27] is used in many sensor fusion implementations [19,25,26]. However, the low resolution of the sensor limits its functionality in object detection systems. Other approaches use stereo event camera systems in combination with high-resolution frame-based stereo systems to calculate depth in the 3D coordinate system [18,20,22]. The implementation of a multi-modal stereo system where image points are projected between event-based and frame-based image planes of contrasting resolutions is limited to [24]. This work shows interesting performance improvements that can be made by such a system. However, the effects on the complexity of the resulting network are not evaluated.

### 2.3. Foveal Vision

The idea of replicating the human biology of the eye has been incorporated into imaging technologies in recent years in an attempt to minimize the trade-off between temporal resolution and spatial resolution. Constandinou et al. explore this in [28] by implementing an ‘adaptable foveating vision chip’ that uses photoreceptors to dynamically determine areas of the image that would benefit from high spatial resolution. The sensor uses macropixel blocks in the periphery, while the pixels are used individually in the foveal regions to give a high spatial resolution. This combats the trade-off between the spatial and temporal resolution of images, as the peripheral areas have a lower resolution, allowing for a much higher temporal resolution to be achieved.

This idea is also developed in [29], which implements a neuromorphic image chip where the spatial resolution varies across the pixel array. A feature detection algorithm is used to determine the key points in the image frame that are used as the center of the visual field, giving the highest resolution areas in the chip. The image resolution then decreases concentrically, moving away from these points to maintain the accuracy of the region of interest while minimizing bandwith. The chip was tested on singular images, where a fixed foveal area was used to reduce network bandwidth. The use of dynamic foveation in [28] makes it much more suitable for use in monitoring systems; however, the specifications of the chip, with a temporal resolution of 2 kHz in the high temporal region, could be further improved with the use of an event-based camera. According to the PROPHESEE website [2], its Metavision event sensor can achieve a temporal resolution greater than 10 kHz.

The ideas of artificial foveation explored in [28,29] could be further developed to investigate the improved accuracy of object detection in the resulting images. Butko and Movellan implement a digital fovea approach in [30], with the aim of improving the efficiency of system object detection. Each image is scanned using a model based on the movement of the eye to determine the area of the image most likely to contain an object. This is used as the high-resolution foveal area, and similar to [29], the resolution decreases in concentric steps moving away from this point. The results show impressive latency improvements with a limited loss in accuracy; however, the evaluation is limited to images only and the implementation is limited to only one foveal region per image. This would make it difficult to detect two objects located on opposite sides of the image, as only one of the objects would be in the high-resolution foveal region.

Rather than reducing the resolution of peripheral regions, Thavamiani et al. propose a foveal vision approach to real-time object detection by magnifying areas of video frames that are likely to contain objects in [31]. A Kernel Density Estimation (KDE) saliency generator is used to generate a saliency map for each video frame from the detections made in the previous frame. The resulting map is used to warp the image, magnifying the salient areas where objects are likely to be. The results are compared against a baseline where the frames are down-sampled, showing improved object detection accuracy, particularly for smaller objects located in these regions. This accuracy improvement comes at a latency cost, as the pre- and post-processing steps involved in the magnification, as well as the saliency map generation, add additional processing time to the system.

The improvement of object detection accuracy often comes at the cost of increased latency. The idea of foveal vision aims to improve the efficiency of imaging systems by improving accuracy, while maintaining low power and latency. The implementation of foveating sensors, where the sensor resolution changes across the pixel array, is investigated in [28,29], where the authors use photoreceptors to detect luminosity changes. Artificial foveation object detection shows improvements in latency in [30] and accuracy in [31] through image scanning and processing. An event camera is a neuromorphic sensor based on human vision and therefore would make an interesting addition to these artificial fovea implementations.

### 2.4. Summary

This review investigates the use of neuromorphic image systems such as event-based cameras and foveal vision to improve the efficiency of object detection systems. The advantages of these neuromorphic sensors over regular frame-based cameras show impressive improvements in imaging applications such as SLAM and object detection in adverse weather conditions. However, the low-resolution images provided by these sensors cannot achieve the same accuracy as state-of-the-art frame-based cameras.

The recent literature has implemented the sensor fusion of event-based cameras with frame-based cameras to combine the advantages of each camera; however, this often increases the complexity and power requirements of the systems. With the adaptation of some previously proposed camera calibration techniques, the sensor fusion of an event camera with an RGB camera can be designed to implement a digital fovea system to mimic the behavior of the human eye and provide further accuracy and efficiency improvements over previous implementations.

With this design, the gaps in the current research can be reduced to investigate whether the high dynamic range and temporal resolution of a neuromorphic camera can be utilized to develop an accurate real-time object detection system while maintaining the low latency and energy consumption characteristics of the asynchronous event camera.

## 3. Methods

### 3.1. Calibration

Camera calibration establishes the relationship between points in the real, 3D world and their corresponding locations on the 2D image plane captured by a camera. By determining the intrinsic and extrinsic parameters of the camera, such as focal length, principal point, lens distortion, and the camera’s position and orientation in space, the calibration process enables accurate mapping between 3D real-world coordinates and 2D image coordinates. These camera parameters are needed to project pixel coordinates from the event camera image plane to their corresponding coordinates in the RGB camera image plane.

The standard method of camera calibration, proposed by Zhang in [21], involves taking pictures of a planar checkerboard pattern from multiple different angles. The corners of the checkerboard are then detected by the calibration algorithm and reprojected using the intrinsic and extrinsic parameters of the camera. This method had to be adjusted to calibrate the event camera, as a planar checkerboard would not trigger any events in the sensor. To combat this, a flashing checkerboard GIF (Graphics Interchange Format) was created, and the cameras were calibrated using the following steps:

1. Create flashing checkerboard GIF and display on large screen.
2. Mount RGB camera on apparatus beside event camera and ensure apparatus is stable and secure.
3. Record video of flashing checkerboard using both cameras simultaneously and repeat for 10–20 different camera angles.
4. Extract singular frame from each video, and extract image from each frame.
5. Process images to improve clarity and ensure image sizes are equal.
6. Calibrate cameras using calibration software to determine the intrinsic and extrinsic parameters of each camera.

**Figure 3:**
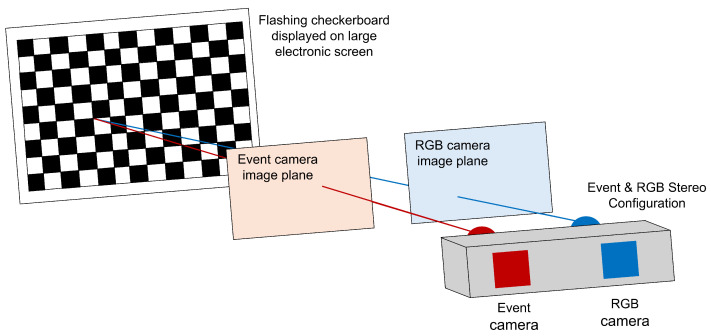
Camera setup used to calibrate the event camera with the RGB camera.

Figure 3 shows the calibration setup. The screen used is mounted on wheels, allowing it to be moved into different positions to collect images at different angles. The image planes depicted in the figure highlight the need for camera calibration, showing that the checkerboard corner is at different locations in the image of each camera.

#### 3.1.1. Calibration Software

**Figure 4:**
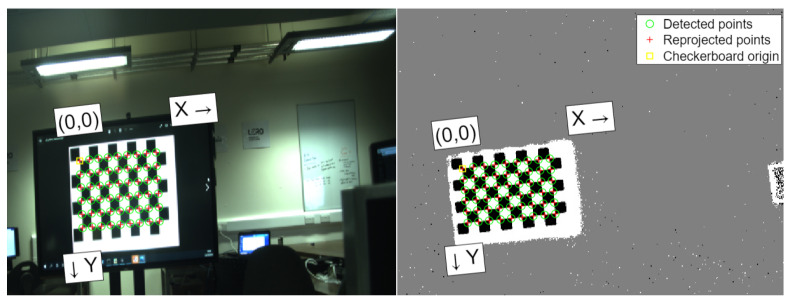
Projection results for the RGB camera (**left**) and event camera (**right**) using the Matlab Stereo Camera Calibrator. The checkerboard corners automatically detected by the software are marked in green, and the reprojected points determined using the stereo calibration results are shown in red.

The Matlab Stereo Camera Calibration application was used to calibrate the two cameras. The RGB images were resized to match the resolution and aspect ratio of the event camera. Corresponding images from the event camera and the RGB camera were analyzed by the Matlab stereo calibration application. The camera parameters were estimated using the algorithm proposed by Zhang in [21] to give the projection results shown in Figure 4. Furthermore, the relative positioning between both cameras remained constant. They were mounted on aluminum extrusion, ensuring that their relative positions did not change between calibration and data capture. The impacts of temperature variations and mechanical vibrations were negligible.

**Figure 5:**
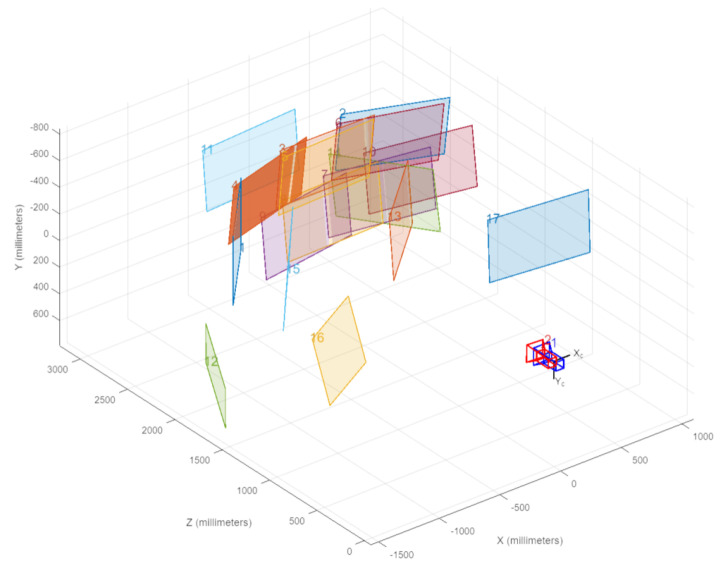
Camera-centric diagram taken from Matlab. The stereo camera pair is shown (event camera in red, RGB camera in blue), and the relative locations of the checkerboard for each position used in the calibration (numbered 1–17).

Figure 5 shows the camera-centric diagram generated by the Matlab R2023a Stereo Calibration tool. It shows the stereo camera pair and its real-world position relative to each of the checkerboard images used in the process. The overall mean error achieved for seventeen images was 0.24 pixels, with a range of [0.09, 0.42]. In general, a mean error of <1 pixel is acceptable; therefore, all images meet this requirement and can be included in the calibration.

Further information on the calibration process can be found in Appendix A.

#### 3.1.2. Translation of Points

To implement the projection of image points from event camera coordinates to RGB camera coordinates in Python (Python rev 3.8, numpy rev 1.23.1), the reprojection process must be converted to an equation that can be used in a Python script. Equation (1) represents the transformation from world coordinates, [X, Y, Z], to camera image points, [*x*, *y*]. Where *R* is the 3D rotation matrix, *t* is the translation vector, *s* is a scalar, and *K* is the intrinsic matrix described in Equation (2), where $f_{x}$,$f_{y}$ and $pp_{x}$, $pp_{y}$ are the x-y focal lengths and principal point, respectively.

(1) $$s\;\cdot \;\left[\begin{array}{c} x\\ y\\ 1 \end{array}\right]=K\left[\begin{array}{cc} R & t \end{array}\right]\;\cdot \;\left[\begin{array}{c} X\\ Y\\ Z\\ 1 \end{array}\right]$$

(2) $$K=\left[\begin{array}{ccc} f_{x} & 0 & pp_{x}\\ 0 & f_{y} & pp_{y}\\ 0 & 0 & 1 \end{array}\right]$$

By specifying Equation (1) for both cameras, and dividing the two resulting equations, Equation (3) is derived, which can be used to project points from coordinates in the plane of camera two, $\left[x_{2},y_{2}\right]$, to coordinates in the plane of camera one, $\left[x_{1},y_{1}\right]$ by assuming the points are at infinity.

(3) $$\frac{s_{1}}{s_{2}}\;\cdot \;\left[\begin{array}{c} x_{1}\\ y_{1}\\ 1 \end{array}\right]=\frac{K_{1}\left[R_{1}t_{1}\right]}{K_{2}\left[R_{2}t_{2}\right]}\;\cdot \;\left[\begin{array}{c} x_{2}\\ y_{2}\\ 1 \end{array}\right]$$

The scalar value $\frac{s_{1}}{s_{2}}$ was found to be 1.0 ± 0.01 for each set of parameters, and therefore rounded to 1 and ignored in the calculation. Equation (3) was tested in Matlab to project the checkerboard corners from one camera image plane to the other, and the results were the same as those achieved by the calibration software. This showed the validity of the equation to be used to translate coordinates from the event camera plane to the RGB camera plane.

#### 3.1.3. Average Extrinsic Parameters

To calculate the reprojection error, Matlab software uses the estimated intrinsic and extrinsic parameters to project the checkerboard corner coordinates from world coordinates to image coordinates. The intrinsic parameters of a camera are constant; however, the extrinsic parameters determine the location of the camera in the 3D world and, therefore, change depending on the location of the camera. In this projection, the checkerboard pattern is used as the ground plane in the world coordinate system, and the extrinsic parameters are calculated relative to that ground plane, giving a different set of values for each checkerboard angle. When investigating the projection using Matlab functions and Equation (3), the extrinsic parameters calculated for each checkerboard angle were used in the projection, therefore changing the equation for each image. This reprojection method could not be used on the collected data, as the extrinsic camera parameters were not measured during data collection.

For each checkerboard angle, the extrinsic parameters of each camera differ due to the changing location of the cameras relative to the pattern. One thing that remains constant over all angles is the position of the event and RGB cameras relative to each other. This position is also constant between the calibration data and the dataset used in the investigation. Equation (4) was used to calculate the division of the extrinsic parameters of one camera by those of the second camera. This average extrinsic value was found to be relatively constant across all images. The average was calculated for all seventeen angles to give a mean value and standard deviations for each matrix point shown in Equations (4) and (5), with an average standard deviation of 0.0046 for all points of the matrix.

(4) $${\left(\frac{R_{1}t_{1}}{R_{2}t_{2}}\right)}_{\mathrm{average}}=\left[\begin{array}{ccc} 1.0039 & -0.0307 & -0.0016\\ 0.0395 & 1.0032 & -0.0032\\ -0.257 & -0.0145 & 0.9985 \end{array}\right]$$

(5) $$\sigma =\left[\begin{array}{ccc} 0.011775 & 0.005389 & 0.00667\\ 0.008242 & 0.003781 & 0.004666\\ 0.000466 & 0.000223 & 0.000268 \end{array}\right]$$

**Figure 6:**
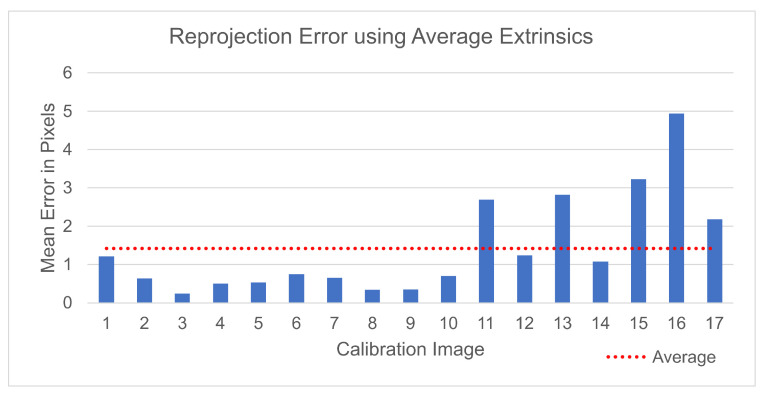
Mean corner reprojection error for each calibration image achieved by projecting detected checkerboard corners in the event camera image to the RGB image plane according to Equation (6).

By incorporating this value with Equation (3), the final projection Equation (6) was determined. This was used to project the checkerboard corners from the event camera image plane to the RGB camera image plane for each of the patterns used in the calibration and the reprojection error results shown in Figure 6 were determined. The projection error shows an increase over the results achieved by the Matlab script with a mean error of 1.42 pixels; however, this method is more flexible and can be used in any situation as long as the cameras remain in the same position relative to each other.

(6) $$\left[\begin{array}{c} x_{1}\\ y_{1}\\ 1 \end{array}\right]=\frac{K_{1}}{K_{2}}\;\cdot \;{\left(\frac{R_{1}t_{1}}{R_{2}t_{2}}\right)}_{\mathrm{average}}\;\cdot \;\left[\begin{array}{c} x_{2}\\ y_{2}\\ 1 \end{array}\right]$$

### 3.2. Fovea Camera Implementation

**Figure 7:**
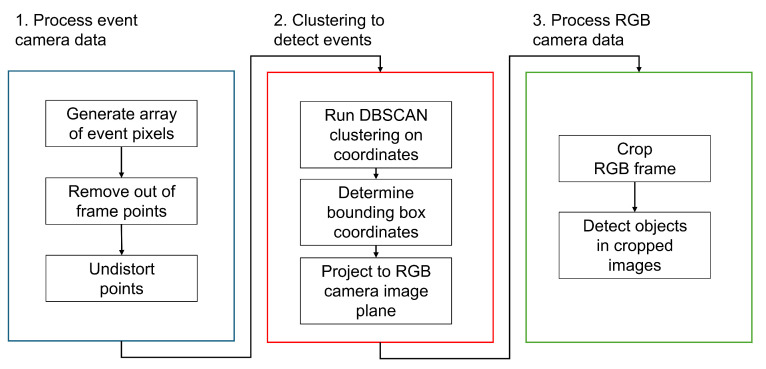
Main steps in data analysis.

The proof-of-concept design uses an event camera and an RGB camera to detect moving objects in a scene according to Figure 7. Moving objects are detected using a clustering algorithm on the event camera data. The intrinsic and extrinsic parameters calculated from the cameras are used to project the bounding box coordinates from the event camera image plane to the RGB image plane using Equation (6). The RGB frame is then cropped using these projected coordinates, and object detection is run on the cropped images.

#### 3.2.1. Data Processing

**Figure 8:**
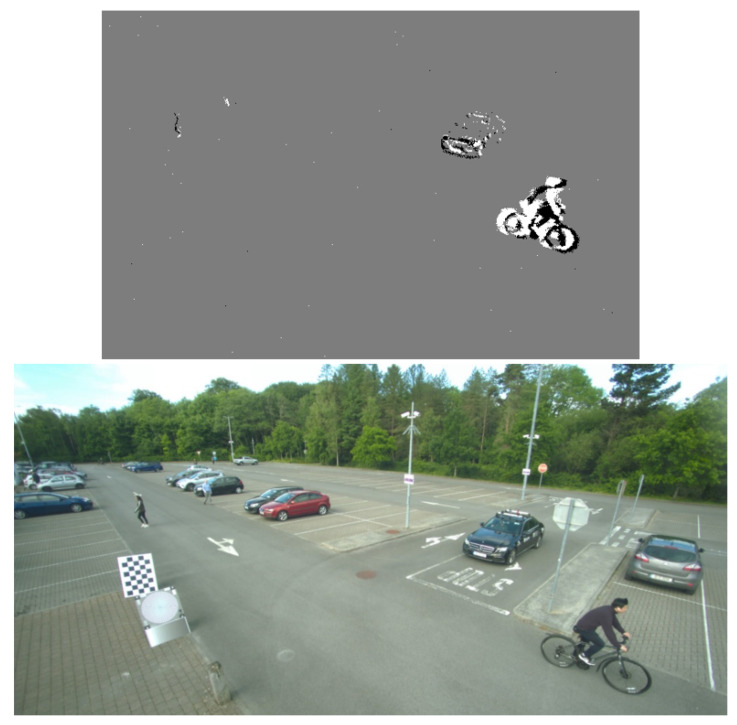
Sample frames from the dataset: The top image is an event camera frame, generated from the CSV file, while the bottom image is the corresponding RGB frame captured by the stereo camera setup.

Before proceeding with system implementation, some initial processing of the data is required. The event data are stored in a csv file. Each row of this file is in the form of [epoch, *x*, *y*, polarity], where the epoch is the elapsed time, in microseconds, at the time of the event, (*x*, *y*) is the pixel coordinate of the event, and the polarity signifies the direction of the change in light intensity. For testing purposes, a thirty-second sample is taken from the data for each camera. Initially, the event camera data are converted into frames that can be used for comparison with the RGB video frames. The RGB data were recorded at a frame rate of 30 fps, giving an accumulation time of 33,333 µs. Frames from the event data are generated using this accumulation time, and synchronized with the RGB video. Figure 8 shows the result of this with the resulting event camera frame, and its corresponding frame from the RGB camera video.

#### 3.2.2. Clustering & Projection

After the visualization of the event camera frames, a video was created and compared with the RGB video at the same time interval. The data were not automatically synchronized during collection; therefore, the videos had to be manually aligned. Manual hyperparameter tuning was also performed on the clustering algorithm to determine the optimal hyperparemeter values of epsilon = 20, minPoints = 10.

**Figure 9:**
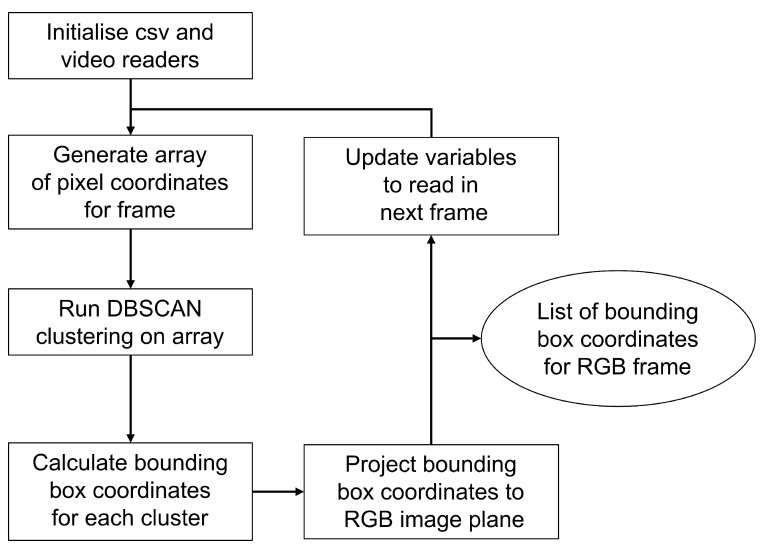
Flowchart highlighting the flow of code to read in the camera data, detect clusters of moving objects, and project points to RGB image plane.

Figure 9 highlights the steps required to read the data for each camera, perform a clustering of event coordinates, and project the resulting cluster bounding boxes to coordinates in the RGB camera image plane for use in subsequent processing steps. The event camera data are read, and an array of pixel coordinates is generated for each frame. This is then inputted into the clustering algorithm to determine the locations of moving objects in the frame. Bounding box coordinates of each object are determined from the resulting clusters and projected to their corresponding pixel coordinates in the RGB camera image plane using Equation (6). These RGB coordinates assume the same frame resolution as used in the calibration.

### 3.3. Object Detection

#### 3.3.1. Performance Measurement

Mean Average Precision (mAP) is a popular evaluation metric for object detection, as it combines precision, recall, and intersection over union (IoU). There are two methods to implement this, both calculating the AP for predictions based on an IoU threshold. If the calculated IoU is above the threshold, it is marked as a positive detection; otherwise, it is marked as negative. The Microsoft COCO (Common Objects in Context) implementation [32] calculates AP over a range of IoU thresholds. The standard COCO metric measures over ten thresholds from 0.5 to 0.95 with an increment of 0.05, denoted as AP50:95; however, this can be adjusted depending on the implementation.

If unspecified, the range of IoU thresholds used in COCO AP is 0.5:0.05:0.95. The second implementation used in the PASCAL VOC dataset [33] calculates the average AP for each class for a fixed IoU threshold. AP50 uses a threshold of 50%, while AP75 uses a threshold of 75%.

Inference Time is the time taken for a neural network to make a prediction and is a key performance measurement in optimizing the efficiency of a system. For the measurement of network latency, the inference time taken by the model to detect/classify objects is recorded for each frame in the analysis. This measurement is repeated for ten iterations, and the average is calculated for each frame.

#### 3.3.2. YOLO Algorithm

YOLO (You Only Look Once) [34] is a single shot object detection algorithm that performs object classification and object localization in the same step. Given the real-time constraints of the system, YOLO is a good choice, as it provides a low inference time. YOLOv5s [35] is used because of its ease of integration with PyTorch (revision 1.8.1), and the small version gives a good trade-off between inference time and AP.

#### 3.3.3. Proof-of-Concept Implementation

The model can be downloaded from GitHub [35], and inference can be run with PyTorch using only a few lines. The resolution requirements for the model are for the input images to be 640 × 640 pixels; however, using PyTorch, all required data augmentation is automatically completed on the image inputted into the model.

**Figure 10:**
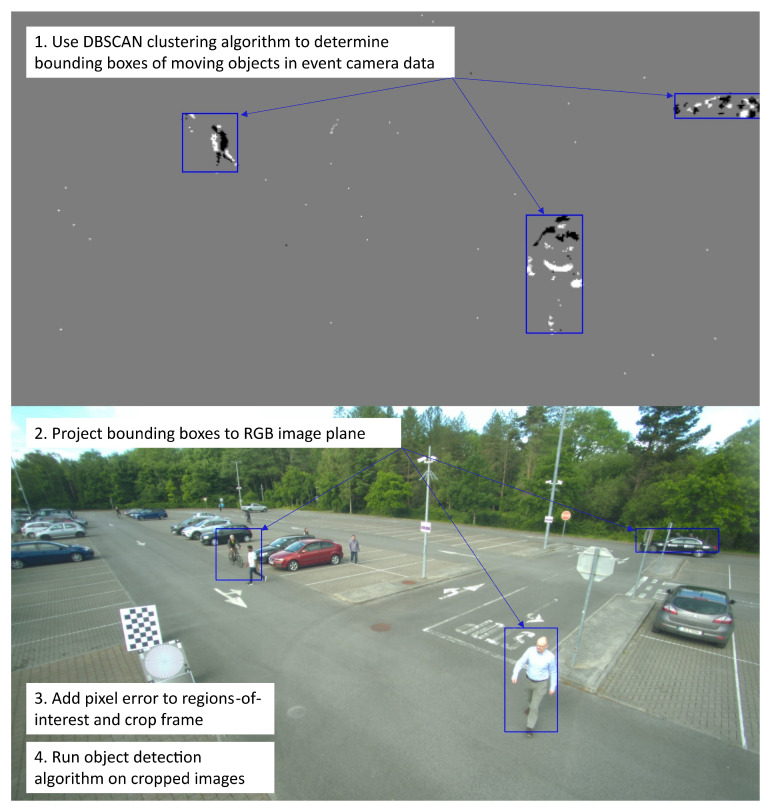
Example frame highlighting steps required for proof-of-concept implementation. The blue boxes in the top image show the bounding boxes determined by the clustering of events on the csv file. These are then projected according to the calibration parameters to give the boxes shown on the bottom image. From here, the RGB image can be cropped prior to inference.

The system is implemented according to Figure 10. The bounding boxes determined by the clustering and projection are used to crop the RGB frame to regions containing moving objects. This eliminates the need to down-sample the image to meet the resolution requirements of the model, therefore reducing or even eliminating any information loss from the high resolution RGB image frame. A pixel error of 100 pixels is added to each dimension of the bounding boxes to ensure that the desired objects are visible in the cropped images.

**Figure 11:**
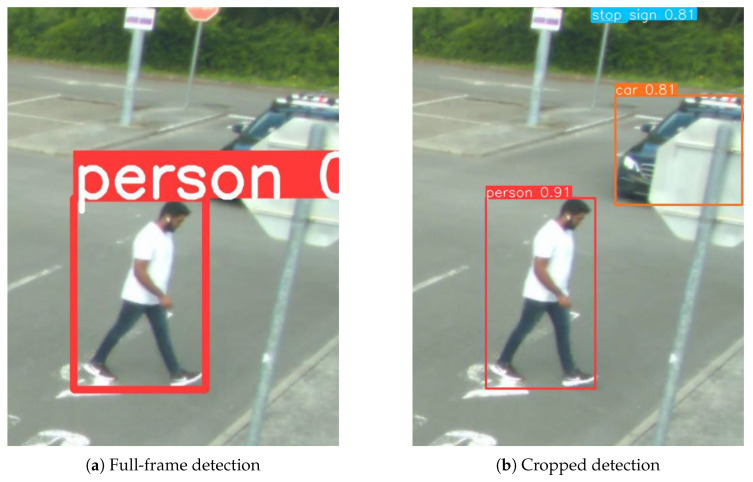
YOLO object detection results. In the cropped image, both the car and stop sign are detected, whereas neither objects are not detected in the full-frame image.

Analysis was performed according to the flow chart shown in Figure 9, and the results were compared with running the YOLO algorithm on the full-frame from the RGB camera to investigate the improvements achieved by cropping the frame instead of down-sampling. Figure 11 shows the detection results for one of the frames, (a) showing the results on the full-frame image and (b) when using the cropped image.

In the cropped image, the person, car, and stop sign in the background are detected; however, in the full-frame image, only the person is detected. The full-frame image has a resolution of 4096 × 2160 pixels, which is downscaled to meet the resolution restrictions of the model. The cropped image has a resolution of 481 × 650 pixels; therefore, little to no downscaling is run on the images, eliminating information loss. The benefits of this are clear from these results.

#### 3.3.4. Data Annotation

To measure the accuracy of the system, target annotations are needed to compare against model predictions. CVAT [36] is an open-source video and image annotation tool for computer vision. The RGB camera video and the generated event camera video were both imported into the software. Moving objects in the RGB video were annotated according to three target labels—person, car, and bicycle—to match the labels of the YOLO model.

To determine the location in the image to start annotating objects, the event camera video was used. Moving objects in the data that are too far away to be detected by the event camera were not annotated. This ensures that the accuracy comparison is based only on object detection and not on the limitations of the camera used.

### 3.4. Event-Region Proposal and Classification Network (EPCNet)

#### 3.4.1. Design Overview

All object detection algorithms, from single-stage algorithms such as YOLO to two-stage algorithms such as Faster R-CNN, involve two processes: object classification and object localization. These give a class prediction and bounding box coordinates for each object in the image. Two-stage algorithms use a region proposal network to locate objects in the image and then run classification on these detected regions.

Single-stage algorithms, such as YOLO, improve the latency of the network by combining these processes into a single step; however, they still need to classify objects and determine their location in the image to give a prediction of the bounding boxes of each. Classification algorithms have only one task; they take in an input image and output the class label of the object in the image, therefore reducing the complexity of the model network.

**Figure 12:**
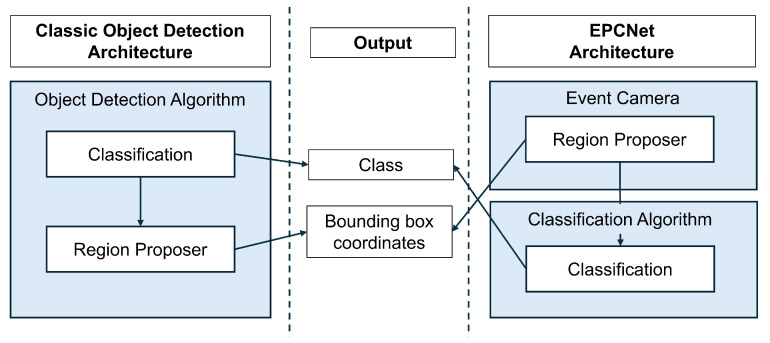
Comparison of the EPCNet architecture (**right**) using the event camera as a region proposer, with the architecture of a classic object detection algorithm (**left**). Both architectures give the same output of object class and bounding box coordinates.

The EPCNet uses an event camera to locate moving objects in a scene in terms of their coordinates on the image plane of the event camera. These coordinates are then projected to the image plane of an RGB camera to give bounding box coordinates of moving objects in the RGB frame. This means that the event camera acts as a region proposer, eliminating the need for an object detection algorithm to do the same thing. Using this idea, a classification algorithm can be run on the proposed regions, reducing the complexity of the network used in the system. Figure 12 highlights this design, showing the same output of class and bounding box coordinates achieved with each implementation.

#### 3.4.2. Classification Model

To implement this, a classification model was trained using transfer learning to classify the three classes used in the data: person, car, and bicycle/cyclist. To train the model, data containing images of pedestrians, cars, and cyclists are needed to make the desired classifications. Images from each class can be extracted from our dataset. However, to ensure that the resulting model is not overfitted to this dataset, another dataset is needed to add variety to the training data.

The BDD (Berkeley Deep Drive) dataset [32] is a large-scale driving video dataset consisting of 100,000 videos taken by cars driving throughout the United States. The videos are recorded in a variety of weather and lighting conditions, and a key frame is extracted from each video and annotated to create a diverse dataset of annotated images. The image labels are recorded in a JSON file, which includes bounding box coordinates and labels for each of the object instances. These labels are used to identify images containing cars, pedestrians, and riders, which are read and cropped according to the corresponding bounding box to give an image that contains only the object of the desired class.

Initially, all instances of the three target classes were saved; however, for many of these, the resulting image was only a few pixels wide and very unclear. To avoid this, the extraction was run again, and only instances with bounding boxes larger than 50 × 50 pixels were saved. This significantly decreased the number of rider and person instances in the dataset, with only 1030 and 1536 remaining after the removal of some invalid images. There was an abundance of car labels; therefore, 2000 were taken so as not to overpower the dataset with car images.

To extract images from our dataset, the original five-minute RGB camera video is used, and the YOLOv5s algorithm is run on each frame. The results of the detection are analyzed to extract the bounding boxes for the detection of labels: car, person, and bicycle. Like the BDD dataset extraction, the frame is cropped according to these bounding boxes to give images containing just the target object. Rider or cyclist is not a class in the YOLO algorithm, so for the bicycle bounding boxes, the height of the bounding box is increased by 50% to include the person in the image as well as the bike. This resulted in 125,000, 12,000, and 3522 images for the car, person, and rider classes, respectively. Some images were removed from the car and person sets due to objects of the wrong label being present, and 3628 images were taken from each to have a relatively even split of images between the three classes.

The resulting images from each of the data acquisitions are split into train, validation, and test splits of 70%, 10%, and 20% of the images in each, respectively. This is performed for each dataset separately according to Table 2, and the resulting data splits are combined to form the final data distribution. The different datasets and classes are divided independently to ensure that they are equally represented in each phase of the training process.

The image data used in training must be processed prior to model training. The images are resized to 224 × 224 pixels and normalized according to the mean and standard deviation of the images in the dataset. The pre-trained model used for transfer learning is ResNet-18, an 18-layer residual network model taken from [37]. The model is initialized with pre-trained weights trained on the ImageNet dataset, and the final layer is adjusted to have three output features, one for each of the target classes. The model is then trained using a cross-entropy loss function, a stochastic gradient descent (SGD) optimizer, and a StepLR scheduler to decay the learning rate over 25 training iterations.

#### 3.4.3. Training Results

**Figure 13:**
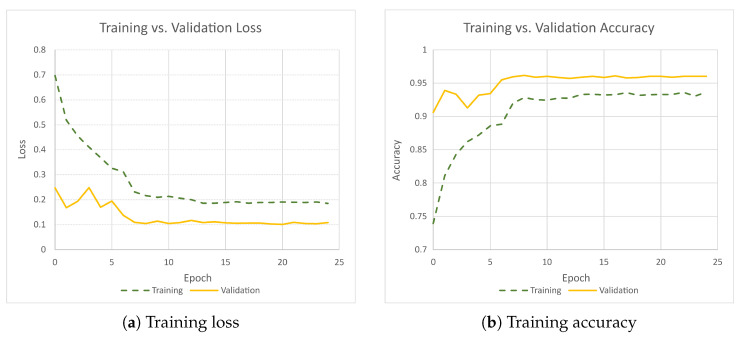
Training metrics across iterations: (**a**) loss convergence and (**b**) accuracy progression.

The accuracy and loss results are highlighted in Figure 13, with the highest validation accuracy of 0.96 achieved during training. It would normally be expected that the validation loss is higher than the testing loss, as the validation data are new data not seen by the network. To investigate this, the original data were resampled to give a different mix of images in each data split, and the model was retrained. This gave similar results, with the model performing better on the validation data than on the training data. A potential reason for this could be that the training data is augmented, while the validation data is not, making the classification task more challenging.

The trained model is then tested on the unseen training dataset, providing an overall precision and recall of 96.4% and 96.5%, respectively. Table 3 shows the precision and recall values for each class and the overall values averaged over all classes; the confusion matrix in Table 4 highlights the predicted vs. actual classes for each image. These results show very high model performance, with the highest accuracy achieved for the car class. The most common misclassification is a person being misclassified as a rider, with 45 of the person images being classified as the rider class. This is the reason for the lower recall for the person class, and a lower precision for the rider class in Table 3.

**Figure 14:**
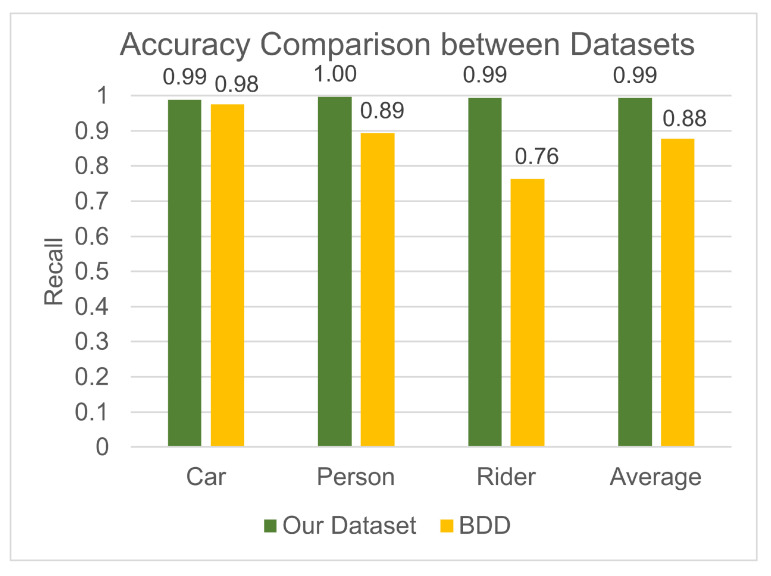
Accuracy of the trained classifier on our dataset, and the BDD dataset. Accuracy is slightly lower for the BDD dataset due to reduced image quality.

To ensure that the model is not overfitted to our dataset, the recall values achieved on each dataset are compared in Figure 14. For the car class, there is very little difference in accuracy between the two datasets. For the person and rider classes, there is a slight decrease in model performance on the BDD dataset, with a 23.1% decrease in recall for the rider class. This does not suggest overfitting, as a slightly lower accuracy is expected due to the lower quality of the images. This lower accuracy is seen for the person and rider classes, but not for the car class, showing the limitations of the BDD dataset for high-quality images of people and riders.

The accuracy results achieved by the model on the test dataset show high performance in all classes, with an overall recall and precision greater than 96%. This trained classification model is used in EPCNet instead of the YOLO object detection model to reduce the complexity of the network, thus providing lower network latency.

## 4. Results

### 4.1. Model Accuracy

Table 5 shows the accuracy results achieved for each class and the IoU threshold for each implementation. ‘FF’ represents YOLO object detection run on each full-frame of the RGB video, ‘Crop’ shows the object detection results achieved when the RGB frame is cropped to regions determined by the event camera, and ’EPCNet’ shows the accuracy results using the bounding box coordinates projected from the event camera and the class label determined by the classification model.

The cropped implementation shows significant accuracy improvements over the full-frame implementation, with the biggest improvement seen for the bicycle class. Using these same IoU thresholds to measure average precision, EPCNet scores poorly with AP results of 11.52%, 34.73%, and 4.38% over the thresholds used in COCO AP, AP50, and AP75.

Analyzing the different classes, the rider and person classes achieve similar results, while the car class shows the highest performance. For a threshold of 50%, the EPCNet performs well on the car class, achieving an AP50 value of 61.93%, scoring higher than the cropped and full-frame implementations.

The limitations of the clustering and projection of bounding box coordinates from the event camera to the RGB camera affect the accuracy calculation for EPCNet. The average IoU calculated for each class were all below 50%; therefore, most detections do not meet the IoU thresholds required for the accuracy measurements used in Table 5 and are not included as correct detections in the evaluation.

To compare the results with a lesser focus on the accuracy of the bounding boxes, Table 6 shows the results for IoU thresholds of 10%, 25%, and 40%. Using these thresholds, more detections are used in the evaluation, as fewer are eliminated due to inaccuracies in the bounding box prediction.

With a threshold of 10%, the EPCNet implementation scores almost the same as the cropped object detection, both with an AP > 65%. This shows a 6% improvement over the full-frame object detection. The EPCNet performs the best for the car class, giving an AP > 70% for each of the three thresholds, scoring higher than both cropped and full-frame object detections for each.

There are limitations to the accuracy measurement of the car class in both object detection implementations, which gives them a lower score. As a result, they cannot be accurately compared, but an AP of 70% shows good performance of the classifier on the car class. The cropped object detection performs the best on the person and car class for all thresholds, but the EPCNet also scores higher than the full-frame object detection on the bicycle/rider class for thresholds of 10% and 25%.

**Figure 15:**
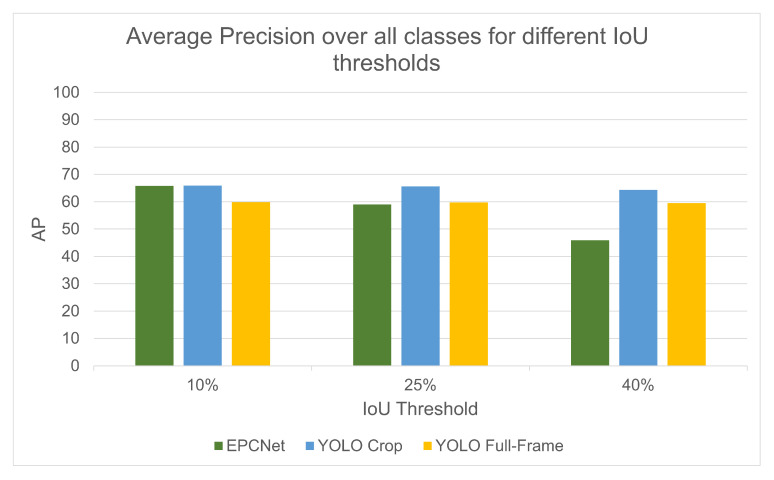
Average precision over all classes for each implementation, with low IoU thresholds of 10%, 25%, and 40%. The EPCNet performs better than the full-frame baseline for an IoU of 10%, and only slightly lower for an IoU of 25%. This shows that the accuracy of this implementation is limited by the projection of the bounding boxes which reduces the IoU.

Figure 15 shows a comparison of average precision scored for each method for all classes, using the same IoU thresholds. The EPCNet performs better than full-frame detection for a 10% threshold, and they perform almost equally at a threshold of 25%. The limitations in the bounding box prediction by the event camera are then clear by the reduction in AP of the EPCNet when the threshold is increased to 40%.

**Figure 16:**
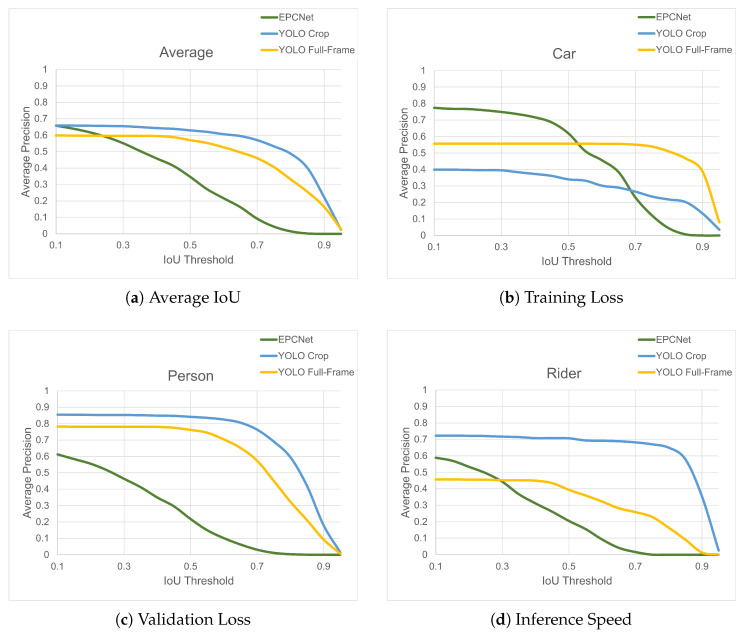
Comparison of average precision for each implementation over IoU thresholds from 10% to 95% for each class. EPCNet performs well for low IoU thresholds, where bounding box prediction has less effect on the IoU.

The effects of increasing the IoU threshold on the average precision of each class for the three implementations are illustrated in Figure 16. For both object detection implementations, the curve remains quite flat up to a threshold of about 0.5, where it begins to decrease, with a sharper decrease at higher thresholds. For person and rider classes, the accuracy of cropped object detection is maintained over a higher range of thresholds than the full-frame implementation, showing improved bounding box detection. This is most prominent for the rider class, where the accuracy is maintained well for the cropped implementation up to a threshold of around 0.8, where it then sees a sharp decline.

For EPCNet, the relationship between accuracy and IoU threshold is almost linear, with accuracy decreasing every time the threshold is increased. For the rider/bicycle class, the classifier achieves a higher AP than the full-frame detection up to a threshold of almost 30%, although it does not match the accuracy of the cropped detection at any threshold.

The EPCNet does not perform well on the person class compared to the object detection implementations. However, for the car class, it performs very well, with an AP above 60% up to a 50% IoU threshold. This shows the potential of the EPCNet if the classification accuracy of the rider and person classes could be improved during the training phase.

Additional information on the accuracy of the EPCNet can be found in Appendix A.

### 4.2. Network Latency

**Figure 17:**
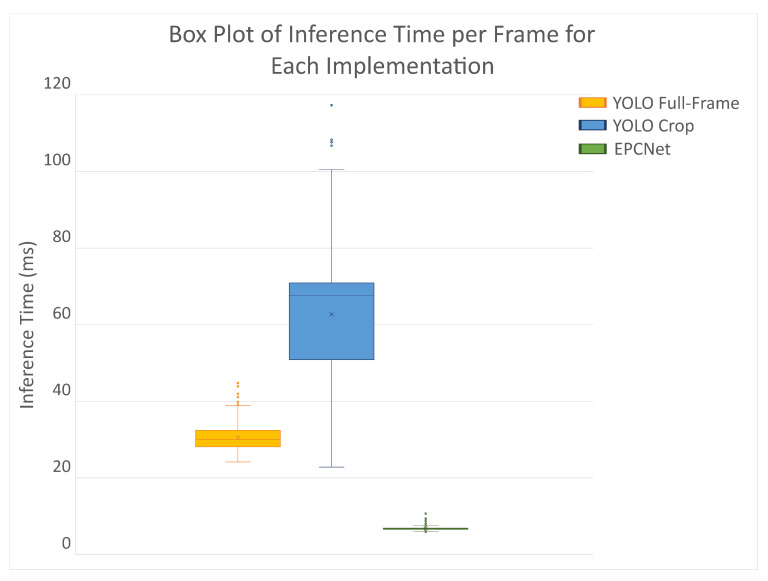
Spread of inference time per frame for each implementation.

**Figure 18:**
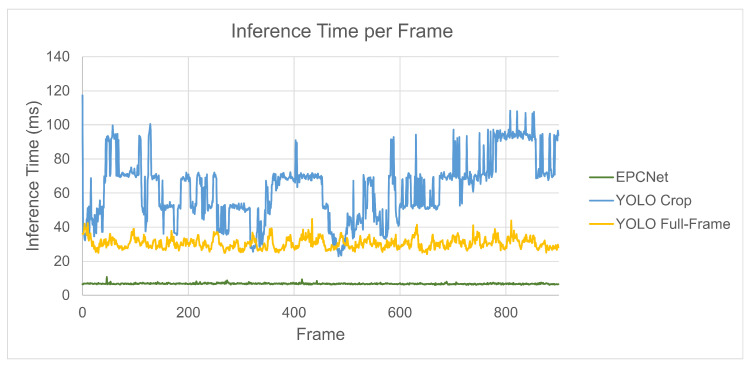
Comparison of inference time achieved for each frame using each of the three implementations.

Figure 17 and Figure 18 show the inference times taken by the models to perform detections and classifications for each implementation. Figure 18 shows the inference time for each of the 900 data frames, while Figure 17 shows the distribution of inference times per frame. The box plot highlights the spread of inference times, including the median, interquartile range, and any potential outliers, providing insight into the variability in processing times across frames.

The standard method of YOLO object detection run on the full-frame RGB data achieves a mean inference time of 30.56 ms per frame, with a standard deviation of 3.16 ms. Due to the increased number of images per frame, the latency doubles for the cropped implementation giving a mean of 62.72 ms with a spread of 18.48 ms. The spread for this implementation is larger because each frame has a different number of objects, resulting in a different number of cropped regions on which to run inference. Although this implementation required object detection to run on 2–6 images for every frame, the inference time increases by only a factor of 2. From Figure 18, the number of cropped images can be estimated based on inference time, and some frames give lower results than full-frame detection, despite having at least two images per frame.

For the EPCNet, the inference time is significantly reduced. The mean inference time for this model was 6.8 ms with a spread of 0.35. Even with multiple images to classify, the mean and spread remain low as image classification is much faster than object detection.

## 5. Discussion

### 5.1. Projection

One of the main challenges faced in this research was the accurate projection from the event camera pixel coordinates to their corresponding coordinates in the RGB camera image plane. A flashing checkerboard pattern was used to calculate the intrinsic and extrinsic parameters of each camera. Using the Matlab Stereo Camera Calibrator, the two cameras were calibrated to achieve an overall mean reprojection error of 0.24 pixels, matching the error achieved by Muglikar et al. in [38], without the use of expensive image reconstruction.

Even after accurate calibration of the two cameras, the projection of points from the event camera to the RGB camera on the pre-recorded dataset posed another challenge. The extrinsic parameters calculated during the calibration process are different for each of the checkerboard angles, due to the location of the cameras changing relative to the checkerboard. This meant that this method could not be used on any pre-recorded data with unknown extrinsic parameters.

The calibration data were recorded by the same camera rig used in our dataset, where the event and RGB cameras remain in a static position relative to each other. This suggests that the relative extrinsics between the cameras would remain constant. With this in mind, an equation was derived to project pixel coordinates from one camera image plane to the other using the intrinsic parameters of each camera, and the average division of the extrinsic parameters of each camera. Using this method, the average reprojection error increased to 1.24 pixels; however, it provided a generalizable approach to the projection of points, which could be integrated into the overall system on the pre-recorded dataset.

This reprojection method could be improved by solving the point correspondence problem between the two cameras. The most accurate method would be to use epipolar geometry, which involves significant calculation and analysis of the camera system, as well as complex geometric projections. Another limitation of the projection when implemented on the test data is the temporal misalignment between the RGB frames and the generated event frames. The timestamps between cameras were not synchronized, and the resulting frames had to be manually synchronized by visual analysis.

### 5.2. Detection & Classification

The accuracy improvements achieved by the design show the benefits of cropping images rather than downscaling for use in convolutional neural networks with resolution limitations. The proof-of-concept implementation improved the COCO AP from 37.93% to 46.89% over all classes using the YOLOv5s algorithm. The greatest improvements were observed for smaller objects, in particular the bicycle class, where the COCO AP increased from 21.08% to 57.38% by cropping the frames. This matches the conclusions made by Thavamani’s artificial fovea implementation in [31]. The improvements of cropping the frames are also evident when comparing the results achieved with AP50 and AP75. For bicycle and person classes, the degradation in accuracy between AP50 and AP75 is more severe for full-frame detection than for cropped implementation, emphasizing the improved bounding box prediction achieved by cropping the RGB images.

The car class shows the opposite, with the AP decreasing for the cropped image for all IoU thresholds compared to the baseline. The accurate measurement of the performance of the model in this class posed a challenge because the model detected all cars in the frame, not just the moving ones. The effects of this were minimized by applying a blurred mask to the images to blur out the static cars.

Despite this limitation, the AP over all classes still shows a substantial improvement over the full-frame baseline. The other important factor when evaluating object detection efficiency is the network latency of the model used. The full-frame implementation gave an average model inference time of 30.56 ms per frame.

For the proof-of-concept implementation, the cropping of the frames results in multiple images input into the model for each frame, which caused the average inference time to increase to 62.72 ms per frame. The COCO AP for the bicycle class increases by a factor of almost two, with the inference time only increasing by a factor of 1. However, for the other classes, the improvement is not as significant; therefore, this increased latency is not validated.

Frames with more cropped regions resulted in a higher inference time, because the model runs detections on more images. This shows the limitations of the method for scenes with many moving objects, as some frames gave inference times above 100 ms. To minimize this, while still maintaining the accuracy improvements, the hyperparameters of the clustering algorithm could be adjusted to create larger clusters, thereby grouping more objects into singular clusters. This would reduce the number of cropped regions, while still maintaining the reduced information loss; therefore, reducing the model latency while maintaining the accuracy improvements.

Another solution to this increased inference time is the use of a classification model proposed by EPCNet. The use of the event camera in the proof-of-concept implementation opens the possibility of using a classification model rather than an object detection model, as the region proposal is run using clustering algorithms on the event camera data. This decreased the model inference time to an average of only 6.8 ms per frame, equivalent to 147 fps, which is more than four times the baseline of 33 fps, or 30.56 ms per frame. The inference time showed little fluctuation between frames, with all latency times below 10 ms despite the increase in the number of images in certain frames. This shows the striking improvements of classification over object detection algorithms when it comes to model inference time.

Similarly to the object detection implementation, the trade-off between accuracy and latency is evident with EPCNet. Compared with object detection results achieved using full-frame and cropped implementations using COCO AP, AP50, and AP75, the EPCNet performs poorly. This is largely due to the previously discussed limitations of the projection of bounding box coordinates, which affect the intersection over union used in the accuracy measurement. Furthermore, the clustering algorithm and hyperparameters used result in multiple objects being grouped into the same cluster; therefore, the resulting bounding box is much larger, and only one of the objects is predicted by the classifier. This results in a low IoU with ground truth annotation.

To minimize the effect of the bounding box prediction on the accuracy results, the AP is compared across lower IoU thresholds. With a threshold of 10%, the EPCNet performs as well as the cropped object detection, and better than full-frame detection, showing that the classification accuracy is improved, but the bounding box prediction is limited.

The availability of pre-trained classifiers is another limitation of this implementation. The model used was trained using transfer learning, but the model accuracy for the rider and person classes was limited by the training data available. The classification model is seen to perform much better for the car class with an AP greater than 60% up to an IoU threshold of 50%. This shows the potential of the model for similar classification results for the other classes with better training data.

By investigating the clustering methods used through hyperparameter tuning and the comparison of algorithms, the grouping of multiple objects into the same cluster could be reduced. The bounding box predictions could be further improved by solving the point correspondence problem, and synchronizing the two cameras to reduce the temporal misalignment. By implementing this and improving the accuracy of the classification model on the person and rider classes through advanced training, the use of the event camera combined with a classification model could give accuracies comparable to the cropped object detection, with significantly lower inference times.

### 5.3. Proposed Design

The latency improvements achievable using the EPCNet have been highlighted; however, the power improvements are also significant when integrated into a complete monitoring system. The power benefits of the event camera over the RGB camera are remarkable, with a power efficiency of only 26 mW, while the RGB camera is 3 W.

The proposed solution is for the RGB camera to be switched off by default; this saves wasted power while there is no movement or changes in the scene. Using the event camera to detect any changes in the scene, its high temporal resolution can be leveraged to quickly detect movement and send a signal to the RGB camera to switch on and record data, where the analysis discussed in this project will commence. The high temporal resolution of the event camera, and the low complexity of clustering algorithms over CNNs will result in the fast, low power tracking of objects, and by using a classification algorithm on the resulting RGB regions, the network latency can be kept to a minimum while avoiding information loss in the images.

The power consumption of the RGB camera can also be further reduced by specifying the target pixels in the image prior to reading it from the sensor. Many sensors allow for a reduction in resolution of the image readout, where information is read only from a subsection of the overall pixel array. By specifying the target pixels in the RGB camera plane according to the regions proposed by the event camera, the image can be read out from only these target pixels, thereby increasing the frame rate and decreasing the power consumption of the RGB camera.

Each of these characteristics shows the potential of the design for real-time systems, with the low power consumption opening up a wide range possible of embedded applications. Currently, the cost of event-based cameras is significantly higher than that of RGB cameras. As event cameras become more common, this price should reduce, making this design more viable.

Although similar sensor fusion approaches between frame-based and event-based cameras have been explored in the recent literature to take advantage of the robustness of event cameras to motion blur and adverse weather conditions, the power advantages have not been fully exploited. By implementing some slight improvements on this design, and by integrating this analysis into an overall system, there is incredible potential for a low-power, high-accuracy real-time embedded monitoring system with robustness to adverse lighting and motion blur.

## 6. Limitations

### 6.1. Initialization

ECPNet uses an event camera to trigger an RGB camera when an object of interest appears in the scene. A key challenge is that machine vision systems require initialization time, which could cause them to miss the target object. However, event cameras offer a solution through their microsecond temporal resolution and frame rates exceeding 10,000 fps. This high-speed tracking capability can monitor objects for hundreds of milliseconds, providing sufficient buffer time for the RGB camera to initialize.

To further optimize the system, several approaches can be considered. First, the RGB camera could operate in a low-power standby mode rather than being completely off, significantly reducing wake-up times while minimizing power consumption. For applications where missing the target is unacceptable, a circular buffer approach could be implemented. In this configuration, the RGB camera would continuously record at low resolution and framerate, then switch to high-resolution mode upon receiving the event camera trigger.

### 6.2. Time Synchronization

To provide a proof of concept, time synchronization is performed manually by aligning frames based on shared visual features in the scene, such as roadside curbs or traffic signs. While this approach is prone to human errors, the results demonstrate that it is satisfactory for initial concept validation.

Furthermore, for production systems requiring accurate time synchronization, hardware synchronization triggers based on GPS time should be implemented. For example, a calibrated DGNSS clock can send simultaneous pulses to both cameras to ensure precise triggering, enabling synchronized timestamps between sensors.

In time-sensitive fusion applications, particularly in design domains such as automated systems on highways with high-speed vehicles, temporal synchronization between sensors is crucial. Without proper synchronization, temporal errors manifest as objects appearing in different spatial locations between sensor frames, leading to degraded fusion results. The faster the objects move relative to the sensors, the more pronounced these spatial discrepancies become, ultimately compromising the system’s ability to accurately track and identify objects.

### 6.3. ECPNet Performance

The lower IoU threshold distinguishes classification accuracy from localization precision. ECPNet’s region proposals identify areas in RGB images that contain objects, but do not provide precise localization information. This approach is particularly valuable for early warning systems where detecting object presence is more critical than determining exact position. Several applications benefit from this trade-off between localization accuracy and power consumption, especially in resource-constrained environments like battery-powered IoT devices. In intrusion detection systems, identifying an intruder’s presence takes priority over their exact location. Similarly, industrial safety systems focus on detecting humans near machinery to prevent accidents, while motion-triggered security systems in smart homes activate alarms based on movement detection rather than precise positioning.

In these scenarios, the event camera processing enables real-time response while maintaining classification accuracy sufficient to reliably trigger downstream tasks. The reduced computational requirements make this approach practical for deployment in power-limited environments where traditional high-precision localization would be prohibitively expensive in terms of energy consumption.

### 6.4. Bounding Box Accuracy and IoU Thresholds

While the proposed system demonstrates significant improvements in latency and energy efficiency through the fusion of event and frame-based cameras, it has several limitations that must be acknowledged.

Firstly, the accuracy of the bounding box projections from the event camera to the RGB image plane is sensitive to calibration precision and noise in event clustering. Despite careful calibration procedures, small misalignments can result in bounding boxes that do not precisely overlap with ground truth annotations. This is particularly problematic when evaluating performance using strict IoU thresholds such as AP50 and AP75, where even slight discrepancies reduce measured accuracy. This limitation is evident in our EPCNet results, which show lower precision at standard thresholds (e.g., 34.73% at AP50 and 4.38% at AP75), despite higher classification accuracy under looser thresholds.

To better understand the system’s behavior under these conditions, we report additional performance metrics at lower IoU thresholds (AP10, AP25, and AP40). These thresholds help to evaluate the classifier’s ability to correctly identify object classes in approximately correct regions, even when bounding box alignment is imperfect. For instance, EPCNet achieves >70% AP at IoU = 10% for the car class, which reflects strong semantic localization despite geometric inaccuracies. We recognize that these non-standard metrics are less common in benchmarking but are useful for revealing practical strengths and weaknesses of systems involving multi-modal projections and asynchronous data sources.

Future work will focus on improving the fidelity of event clustering and enhancing temporal alignment between the event and RGB modalities. Additionally, investigating real-time calibration correction methods or adaptive projection strategies could help bridge the current performance gap at higher IoU thresholds, bringing the system’s precision more in line with conventional object detection standards.

## 7. Conclusions & Future Work

The aim of this work was to implement an efficient real-time monitoring system using the sensor fusion of an event camera and an RGB camera to combat the trade-off between accuracy and latency, while minimizing power consumption and maintaining robustness to adverse conditions.

This was first implemented using a foveal vision approach, where the event camera is used to locate moving objects in the scene so that the RGB frame can be cropped instead of down-sampled before input into an object detection algorithm. This approach yielded a significant 8.96% increase in COCO AP across all classes, with the bicycle class notably improving by a factor of 1.72. However, this performance gain came at the cost of increased inference time. To mitigate the information loss caused by aggressive down-sampling, the implementation crops the image into different regions, thereby maintaining higher resolution in these sections. The model is then run on each of these cropped images. While this preserves more information and leads to the higher COCO AP, processing multiple crops per frame inevitably results in a longer overall inference time, highlighting a trade-off between accuracy and speed.

The Event-Region Proposal and Classification Network (EPCNet) is proposed, where the event camera acts as a region proposer, allowing a classification algorithm to be used in place of the object detection model. The accuracy of this implementation is limited by the clustering and projection of bounding boxes; however, using an IoU of 10%, the AP matches that of the cropped object detection. The main advantage of this design is the reduced complexity of the model, which achieved an average network latency of only 6.8 ms per frame, a 78% improvement over the baseline. This design also capitalizes on the advantages of the event camera, producing a low-power system with robustness to adverse lighting conditions.

This research is an initial investigation of the novel artificial foveal methods described in the study. Future work will involve more extensive testing and experimentation to further support the results documented in the research. This will include testing on a broader dataset with more samples, different lighting conditions and test scenarios, and real-time implementations. The further investigation of performance in these scenarios will include comparisons of latency, power consumption, and accuracy with traditional methods.

Additional future works may include conducting an ablation study to investigate the sensitivity of ECPNet to the size of the pixel buffer added to the bounding boxes. The current study employed a 100-pixel buffer to ensure that the entire object was captured within the cropped image, as the temporal alignment between the two modalities was performed manually using shared features. This manual alignment may introduce uncertainties or errors into the ECPNet method. By varying the size of the buffer or removing it entirely, we can analyze the trade-off between accuracy and false positives. This ablation study would provide valuable insights into how the padding affects the system’s precision and robustness. In the case of perfect temporal synchronization between the modalities, the influence of the buffer may lead to the capture of background objects or the merging of closely clustered objects, potentially resulting in increased false detections. Therefore, investigating the impact of the pixel buffer size under various temporal alignment conditions would contribute to a better understanding of ECPNet’s performance and limitations.

This research facilitates a variety of possibilities for future improvements. The most significant results of the research are the latency improvements achieved using the classification model. In future work, the implementation of some minor refinements could considerably improve the accuracy achieved by the implementation. Improving the equipment could increase the accuracy of the projection between the event and RGB cameras. To improve classification accuracy, further experimentation and analysis of the clustering algorithm to separate objects into separate clusters and improved classifier training would show notable improvements.

For EPCNet to be viable in real-time applications, the process of clustering, ROI definition, cropping, and classification would need to be faster than single RGB image analysis. This would require the algorithm to be optimized to reduce image processing time, avoiding any additional latency added to the system. The nature of the design means that latency could also be reduced in other ways by redesigning the physical setup and processing pipeline. The resolution of the camera used could be decreased to improve the frame rate or by specifying the target pixels in the camera prior to readout, as discussed in the previous section. All of this will be investigated further in future research.

Finally, in the context of robustness to adverse conditions, there is a range of different image processing techniques that could be run on the cropped images, such as increasing brightness or contrast in the images to help with object detection in difficult lighting conditions, where the RGB camera would normally have trouble.

## Figures and Tables

**Table 1:** Specifications of the cameras used in the research.

	Event Camera	RGB Camera
Model	PROPHESEE ONBOARD (Paris, France)	Blackfly S (Teledyne FLIR, Arlington, VA, USA)
Spatial Resolution (pixels)	640 × 480	4096 × 2160
Temporal Resolution (fps)	>5000	42
Dynamic Range (dB)	>120	<50
Power Efficiency (W)	0.026	3
Pixel Pitch (μm)	15	3.45

**Table 2:** Data splits used for training, showing distribution between our dataset and the BDD dataset.

Class	Our Dataset				BDD Dataset				Total		
	Train	Val	Test	Total	Train	Val	Test	Total	Train	Val	Test
Rider	2467	352	703	3522	720	103	207	1030	3187	455	910
Person	2573	352	703	3628	1075	154	307	1536	3648	506	1010
Car	2573	352	703	3628	1400	200	400	2000	3973	552	1103

**Table 3:** Classification performance metrics (%) across object classes on test data.

Metric	Class			
	Car	Person	Rider	Average
Precision	98.37	96.54	94.18	96.36
Recall	98.02	94.40	97.06	96.50

**Table 4:** Confusion matrix showing true positives (diagonal) and false classifications (off-diagonal) counts.

Actual\Predicted	Predicted Class		
	Car	Person	Rider
Car	1087	13	5
Person	14	977	21
Rider	8	45	857

**Table 5:** Performance comparison (in %) between YOLO Full-Frame (FF), YOLO Crop, and EPCNet implementations across different metrics.

Class	COCO AP			AP50			AP75		
	FF	Crop	EPCNet	FF	Crop	EPCNet	FF	Crop	EPCNet
Car	47.54	23.55	23.71	55.62	34.05	61.93	53.88	23.59	12.01
Person	45.16	59.73	5.75	76.15	84.19	21.76	45.05	69.08	1.12
Bicycle	21.08	57.38	5.11	39.33	70.61	20.50	22.86	67.00	0.03
Average	37.93	46.89	11.52	57.03	62.95	34.73	40.60	53.22	4.38

**Table 6:** Accuracy comparison (%) between full-frame (FF), cropped, and EPCNet implementations across different IoU thresholds.

Class	IoU Threshold 10%			IoU Threshold 25%			IoU Threshold 40%		
	FF	Crop	EPCNet	FF	Crop	EPCNet	FF	Crop	EPCNet
Car	55.62	39.98	77.42	55.62	39.64	75.95	55.62	37.33	71.65
Person	78.27	85.49	61.18	78.06	85.33	51.38	78.03	84.94	34.88
Bicycle	45.67	72.25	58.87	45.46	71.99	49.54	44.88	70.72	31.12
Average	59.85	65.91	65.83	59.71	65.65	58.96	59.51	64.33	45.89

**Figure A1:**
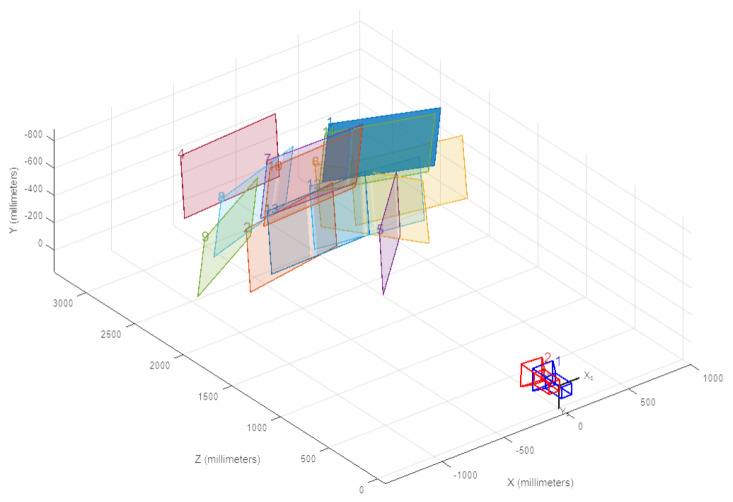
Camera-centric diagram taken from Matlab for the initial calibration. The stereo camera pair is shown (event camera in red, RGB camera in blue), as are the relative locations of the checkerboard for each position used in the calibration (numbered 1–13).

**Figure A2:**
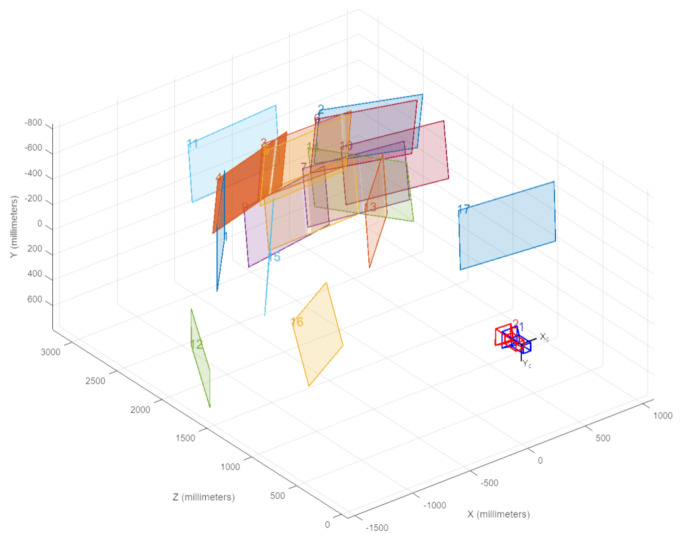
Camera-centric diagram for the second calibration. Note the additional checkerboard angles recorded across the entire FOV of the cameras.

**Figure A3:**
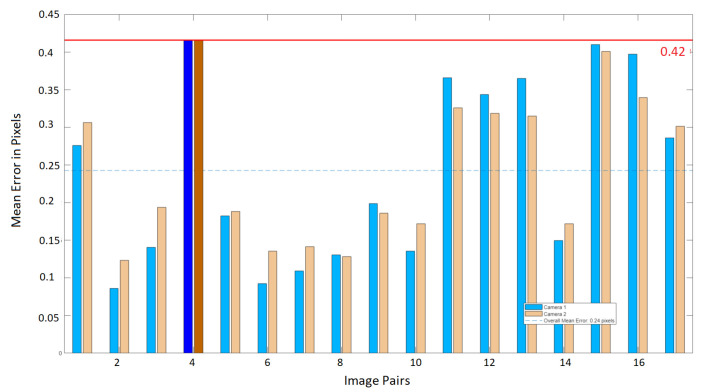
Reprojection errors calculated by Matlab using calibration parameters to project 3D coordinates to image coordinates for the event camera (blue) and the RGB camera (orange). The average error of 0.24 is marked by the dashed blue line and the max error of 0.42 by the red line.

**Figure A4:**
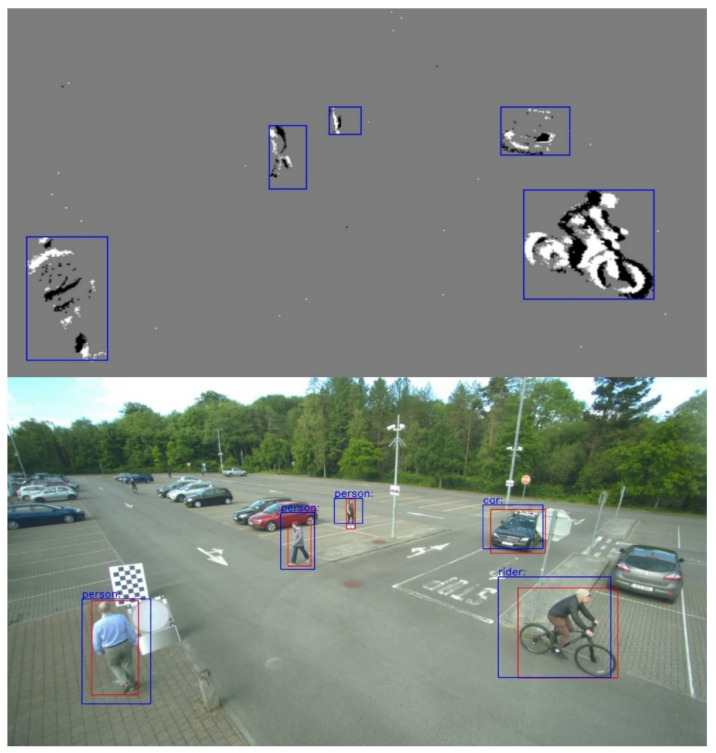
EPCNet results for a sample frame. The event camera frame is shown on top with the event clusters highlighted in blue. The corresponding RGB frame is shown below with the projected bounding boxes and class predicted by the EPCNet in blue. The red boxes show the ground truth bounding boxes for the frame. The EPCNet performs well on this frame, but due to the bounding box projection, the IoU of the boxes is not as good as traditional object detection.

**Figure A5:**
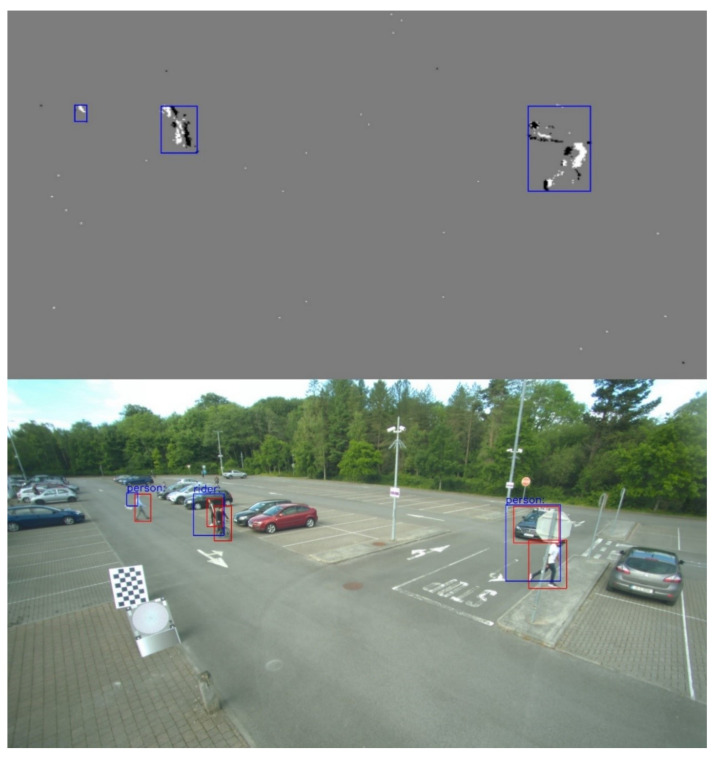
Similar results achieved by the EPCNet on a different frame. Two of the event clusters contain two moving objects. This results in poor average precision for the frame as only one object prediction is taken from the classifier for each ROI.

## Data Availability

The raw data supporting the conclusions of this article will be made available by the authors on request.

## Appendix A

### Appendix A.1. Calibration

There are some points to note regarding the Stereo Calibration of the event camera with the RGB camera. The initial calibration of the cameras gave an overall mean error of 0.09 pixels. The camera-centric diagram from this calibration is shown in Figure A1. Although the reprojection error is low for the images used, the resulting calibration did not give accurate projection results near the edge of the FOV of the cameras, and the stereo rectification of the images was not correctly calculated, suggesting that these calibration results are invalid.

This highlights the importance of accounting for image distortion and including calibration data across the entire FOV of the cameras.

The calibration was repeated to address these issues. The main changes made were as follows:

1. Event camera images were cropped to 640 × 338 pixels to match the aspect ratio of the RGB camera, and the RGB images were downscaled to match this resolution, maintaining their aspect ratio of 1.9.
2. Additional angles were recorded to cover the entire FOV of the cameras and added to original calibration images.

The reprojection result achieved in this second calibration was 0.24 pixels, with a range of [0.09, 0.42] over seventeen images. Comparing these errors with the camera-centric diagram, the errors are higher for the images positioned at more extreme angles, as shown in Figure A2. Excluding image 4, images 2–10 have errors below the mean, and are positioned in front of the camera rig relatively straight on. The other images have higher errors and are positioned at sharper angles, being more spread out across the FOV of the cameras. These images are the reason for the higher reprojection error of this calibration; however, it is important that all angles are covered to ensure that points can be projected across the entire FOV of the cameras. In general, a mean error of <1 pixel is acceptable; therefore, all images meet this requirement and can be included in the calibration.

### Appendix A.2. EPCNet

The results achieved using EPCNet are highlighted in Figure A4 and Figure A5. Figure A4 shows a frame in which the implementation works very well. The clustering algorithm detects separate clusters for each of the moving objects in the frame, with the resulting bounding boxes highlighted in blue on the event camera frame. The RGB frame shows the ground truth bounding boxes in red and the projected bounding boxes in blue. These bounding boxes are used as the region proposals to run the image classification, and the classification results from the model are annotated above each box in blue. Note that these bounding boxes do not include the pixel error added to each region when cropping the image prior to classification by the model.

Figure A5 highlights some of the limitations of the system. There are five moving objects in the image: three people, a rider, and a car. Due to the location of the objects in the frame, the clustering algorithm groups the car into the same cluster as a person, and the rider into the same cluster as another person. The person on the left side of the frame is quite far away from the cameras; therefore, only a few event pixels are detected by the sensor, and the resulting bounding box is very small. The classifier is trained to detect one object per image; therefore, for each image, the prediction with the highest confidence is taken as the result. The model correctly classifies the person in the right region, and the rider in the middle region; however, due to both regions containing two objects, the second object is missed by the model. With the addition of the pixel error before cropping, the person in the left region is correctly identified by the model; however, the accuracy of the bounding box prediction is low. This is due to the limitations of the event camera at that distance, and the accuracy of the projection.
